# Two-step laser synthesis of Ag@TiO_2_ nanomaterials for the photocatalytic degradation of rhodamine B

**DOI:** 10.3762/bjnano.17.43

**Published:** 2026-05-11

**Authors:** Marija Kovačević, Miloš Tošić, Rafaela Radičić, Vladimir Rajić, Nikša Krstulović, Miloš Momčilović, Sanja Živković

**Affiliations:** 1 Vinča Institute of Nuclear Sciences-National Institute of the Republic of Serbia, University of Belgrade, Mike Petrovića Alasa 12-14, 11351 Belgrade, Serbia; 2 Centre for Advanced Laser Techniques, Institute of Physics, Bijenička Cesta 46, 10000 Zagreb, Croatia

**Keywords:** Ag@TiO_2_ nanomaterials, photocatalytic degradation, pulsed laser ablation in liquids, pulsed laser deposition, rhodamine B

## Abstract

This study presents a novel, chemical-free approach for the synthesis of Ag-modified TiO_2_ (Ag@TiO_2_) nanoparticles by combining the two laser-based ablation techniques pulsed laser deposition (PLD) and pulsed laser ablation in liquids (PLAL). Initially, silver was ablated using 200 and 2000 pulses and deposited onto titanium targets via PLD. Subsequently, these modified targets were submerged in water and processed using PLAL to generate the final nanomaterials. Characterization through HRTEM, EDS, and UV–vis spectroscopy confirmed the formation of nanoparticles with a predominantly anatase TiO_2_ phase. The synthesized particles exhibited spherical morphology, with average diameters ranging from 97–331 nm for the 200p sample and 86–144 nm for the 2000p sample. The photocatalytic efficiency was evaluated using rhodamine B (RhB) under UV irradiation; while RhB showed minimal self-degradation, the Ag@TiO_2_ 200p and 2000p NPs samples achieved degradation rates of 75.8% and 88.4%, respectively. The main novelty of this work is the combination of two laser techniques in a chemical-free process, which provides an efficient and environmentally friendly route for the fabrication of Ag@TiO_2_ photocatalysts.

## Introduction

Titanium dioxide has long been recognized as perhaps the most extensively studied photocatalyst owing to its exceptional chemical stability, earth‐abundance, low cost, and benign (non‐toxic) nature [1–3]. These attributes, along with its strong oxidative power and high refractive index, make TiO_2_ an attractive and versatile material for UV–vis photodegradation. In practice, TiO_2_ is widely used in photocatalytic systems for pollutant degradation (e.g., dyes, pharmaceuticals, including drugs, supplements and cosmetics, phenols, and poly(alkyl)acrylates), water purification, CO_2_ reduction, and hydrogen production [2–8]. However, pristine TiO_2_ suffers from intrinsic drawbacks. Its bandgap is large (≈3.2 eV [9]); hence, it absorbs only UV light (a small fraction of sunlight, only 3–4%) and shows very limited visible-light response. Also, photogenerated electrons and holes recombine rapidly [7]. To address this, researchers have developed numerous modification strategies. Silver-modified titanium dioxide (Ag@TiO_2_) nanoparticles have gained significant attention as advanced photocatalysts for the degradation of organic pollutants, particularly under visible-light irradiation. The incorporation of silver introduces localized surface plasmon resonance effects and facilitates the formation of Schottky barriers at the metal–semiconductor interface, both of which contribute to an extended light absorption range and enhanced charge carrier separation. The improved activity is primarily attributed to the narrowing of TiO_2_’s effective bandgap and the suppression of electron–hole recombination, allowing for more effective utilization of the solar spectrum. Accordingly, Ag@TiO_2_ nanostructures, especially those obtained through controlled synthesis, represent highly promising candidates for photocatalytic environmental remediation processes induced by visible light, such as water treatment and degradation of organic contaminants [10–12].

Among various synthesis techniques, laser-based methods, such as pulsed laser ablation in liquids (PLAL) and laser pyrolysis, stand out as clean, efficient, and surfactant-free routes for fabricating Ag@TiO_2_ nanomaterials. These physical processes enable the direct formation of highly pure and well-dispersed nanoparticles from bulk targets without the need for chemical precursors or stabilizing agents. By fine-tuning laser parameters such as wavelength, pulse energy, and ambient conditions, researchers can tailor the morphology, composition, and crystallinity of the resulting nanostructures with high precision. The absence of organic contaminants or residual reagents maximizes the catalytic surface area, while the presence of silver can influence the optical response of TiO_2_ through plasmon-related effects, extending light absorption toward the visible region [12] and enabling efficient activity under visible light. In sum, laser-synthesized Ag@TiO_2_ nanoparticles uniquely combine structural purity and optical enhancement, positioning them as superior candidates for solar-assisted photocatalytic applications [10–17].

**Figure 1:**
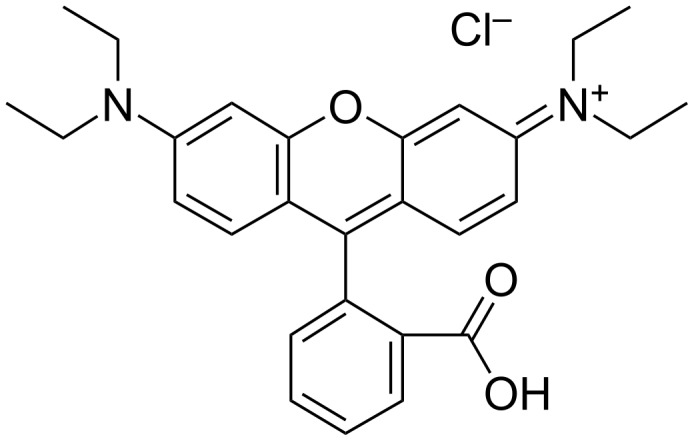
Structure of rhodamine B.

Rhodamine B (RhB, Figure 1) is a synthetic fluorescent dye from the xanthene family, widely utilized across multiple industrial sectors such as textile manufacturing, papermaking, plastic processing, cosmetics, and biomedical applications, due to its vivid color, chemical stability and photostability, and high solubility in water. However, its widespread application and persistent nature raise serious environmental and health concerns. Once released into aquatic environments, RhB resists natural degradation processes, leading to long-term accumulation. It disrupts photosynthetic activity by limiting light penetration in water bodies and causes toxicity to aquatic organisms. Furthermore, RhB is considered a potential mutagen and carcinogen, capable of causing DNA damage and posing significant risks to human health through bioaccumulation in the food chain. Given these factors, RhB is considered a high-priority contaminant, and developing efficient, sustainable degradation technologies for its removal remains a key goal in environmental science [3,18–20].

The main objective of this study was to synthesize silver-modified titanium dioxide (Ag@TiO_2_) photocatalysts via the two steps pulsed laser deposition (PLD) and PLAL. First, using different numbers of laser pulses (200 and 2000 pulses), different amounts of Ag were ablated and deposited on pure Ti targets. Second, in order to investigate how the pulse number influences structural properties and photocatalytic performance, nanomaterials were prepared using PLAL after submerging the obtained Ag-coated Ti targets in water. The efficiency of the synthesized materials was evaluated through the degradation of rhodamine B under UV–vis light irradiation. Comprehensive structural and compositional characterization was performed using scanning electron microscopy (SEM) and transmission electron microscopy (TEM) with energy-dispersive spectroscopy (EDS), aiming to establish a clear correlation between synthesis parameters, nanostructure features, and photocatalytic activity.

In our previous studies [6–7,21–22], laser-based methods were used for the synthesis of metal-modified oxide nanoparticles, showing that the photocatalytic performance strongly depends on synthesis conditions and metal incorporation. While some studies have explored combined laser approaches, these systems were typically based on different materials or did not include controlled metal deposition prior to nanoparticle formation, particularly when using picosecond laser ablation. In this work, we introduce a two-step strategy combining nanosecond PLD and picosecond PLAL. This approach enables controlled silver loading prior to nanoparticle formation, leading to improved control over nanoparticle structure and photocatalytic performance.

## Results and Discussion

### SEM analysis

**Figure 2:**
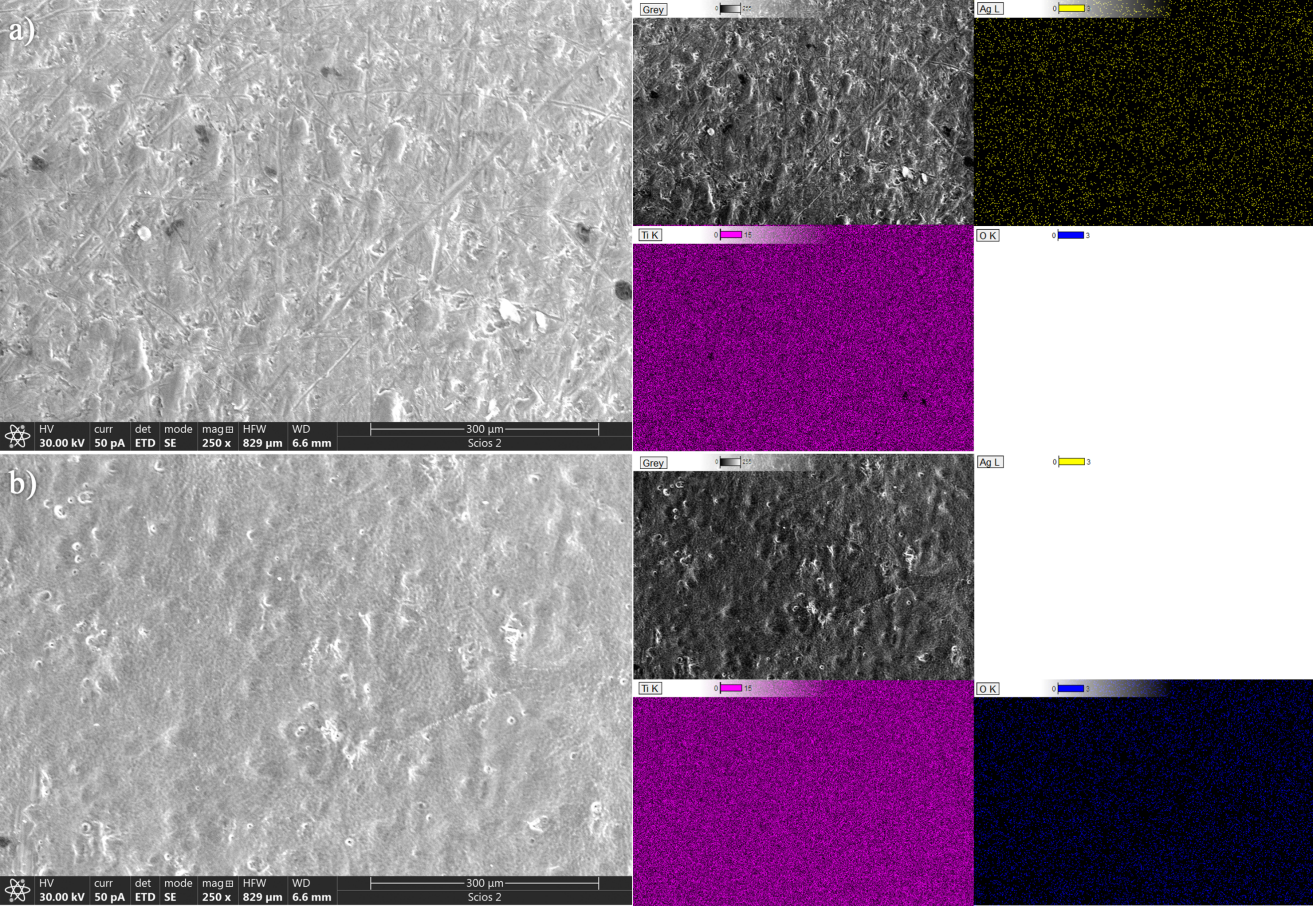
SEM micrograph with corresponding EDS elemental mapping of the Ag@TiO_2_ 200p plate. (a) Surface after deposition of Ag, (b) surface after laser ablation in water.

**Figure 3:**
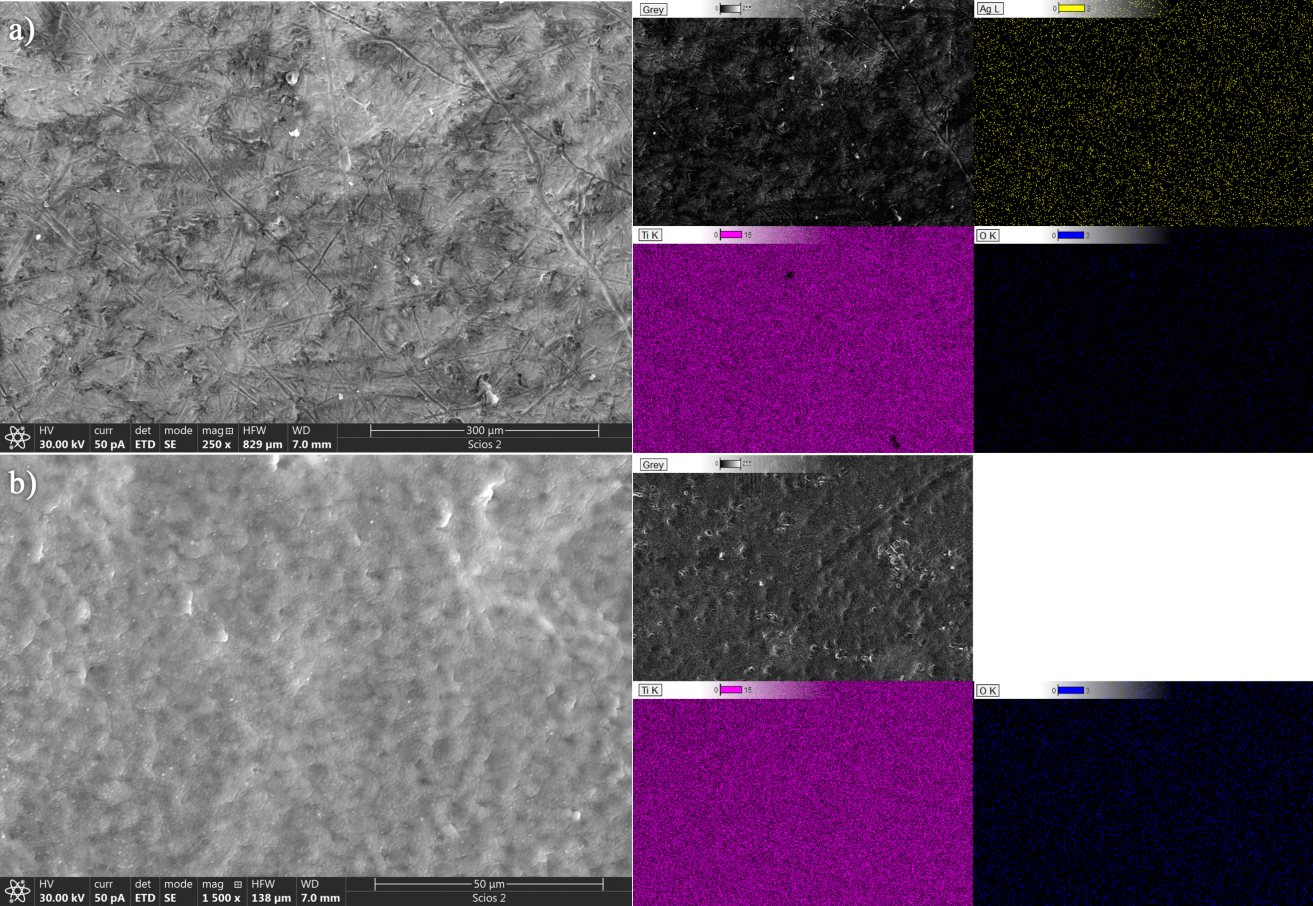
SEM micrograph with corresponding EDS elemental mapping of the Ag@TiO_2_ 2000p plate. (a) Surface after deposition of Ag, (b) surface after laser ablation in water.

The surface morphology of PLD-prepared samples was analyzed using SEM-EDS (Figure 2 and Figure 3). SEM images of the sample surface after deposition of silver on the titanium plate are given in Figure 2a and Figure 3a alongside the distribution of titanium (pink) and oxygen (blue), and silver (yellow) according to the elemental mapping obtained from the EDS analysis. SEM-SE images generated using a focused electron beam to image sample surfaces at high resolution by detecting emitted secondary electrons (SE) for topography showed that there is a small difference in surface morphology between Ag@TiO_2_ 200p and Ag@TiO_2_ 2000p plates. This could be correlated with the silver distribution and concentration on the titanium plate. EDS analysis showed a homogeneous distribution of Ag on both samples with 0.161 and 0.413 wt % of Ag. Additionally, the surface morphology after laser irradiation during the production of nanomaterials is significantly different for these two samples. Even though silver is not detected on the sample’s surface because it is fully ablated, the higher concentration of this metal on Ag@TiO_2_ 2000p affected the ablation rate of the Ti plate and induced a growth of titanium oxide nanostructures on the surface.

### Morphology and size of Ag@TiO_2_ (200p and 2000p)

**Figure 4:**
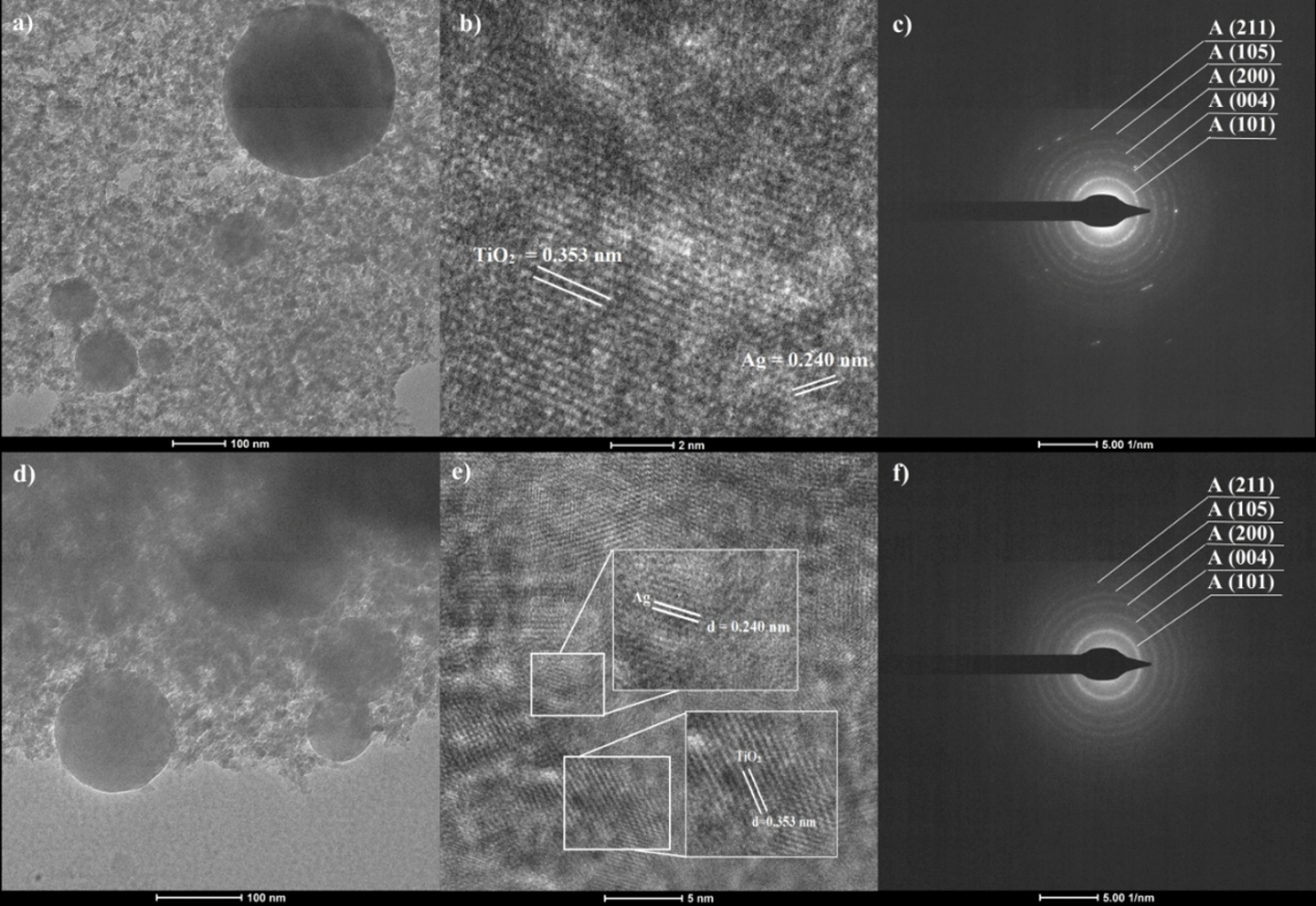
(a, d) Cross-sectional TEM micrographs, (b, e) HRTEM images, and (c, f) SAED diffraction patterns of, respectively, Ag@TiO_2_ 200p and Ag@TiO_2_ 2000p NPs.

Morphology and structure of the obtained samples were analyzed using TEM, HRTEM, and SAED techniques, as illustrated in Figure 4a–f. The TEM images of the Ag@TiO_2_ 200p and Ag@TiO_2_ 2000p nanoparticles (NPs) are displayed in Figure 4a and Figure 4d, respectively. The synthesized nanoparticles exhibit a spherical morphology, with diameters varying from 97 to 331 nm for Ag@TiO_2_ 200p NPs and from 86 to 144 nm for Ag@TiO_2_ 2000p NPs.

**Figure 5:**
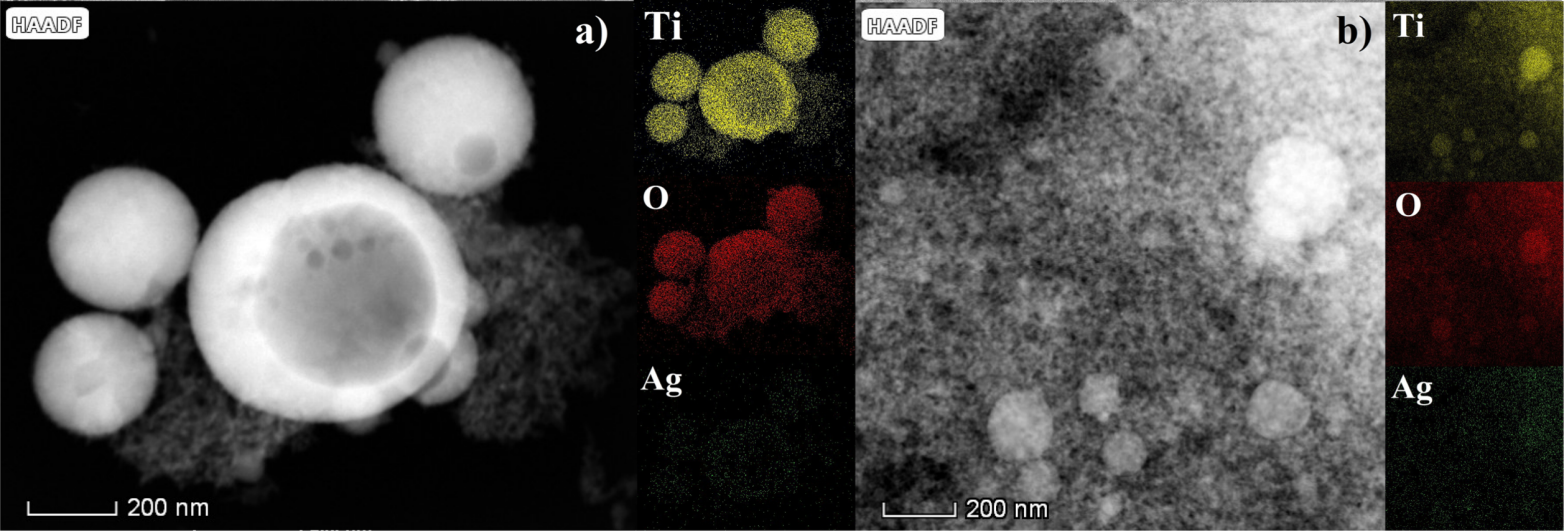
STEM-HAADF images and STEM-EDS compositional maps of titanium, oxygen, and silver of synthesized (a) Ag@TiO_2_ 200p and (b) Ag@TiO_2_ 2000p NPs.

The determined lattice spacings of 0.353 and 0.240 nm, derived from the HRTEM images in Figure 4b,e, can be assigned to the (101) plane of anatase TiO_2_ and the (111) plane of metallic Ag, respectively. The SAED diffraction pattern exhibit concentric diffraction rings, confirming the crystalline structure of the synthesized Ag@TiO_2_ NPs samples. The diffraction rings correspond to the (101), (004), (200), (105), and (211) lattice planes of TiO_2_ nanocrystals (Figure 4c,f). The dominant contribution of TiO_2_-related diffraction rings suggests that the crystalline structure of the samples is mainly governed by the TiO_2_ phase. In addition, mapping pictures of Ti, O, and Ag were collected, as well as STEM/HAADF micrographs of the Ag@TiO_2_ NPs samples that had been manufactured (Figure 5). A regular distribution of titanium (yellow) and oxygen (red) elements can be seen within the TiO_2_ agglomerates, as well as the presence of silver (green), according to the elemental mapping obtained from the EDS. Table 1 summarizes obtained EDS values including the relative error of the applied method. It confirms that Ag is present in these samples, while the mass fraction of Ag is 1.43 times greater in the Ag@TiO_2_ 2000p than in the Ag@TiO_2_ 200p NP solution.

**Table 1:** Atomic and mass fractions of elements in Ag@TiO_2_ nanoparticles obtained by EDS.

Sample	Element	Atomic fraction (atom %)	Mass fraction (wt %)

Ag@TiO_2_ 200p	O K	54.96 ± 7.80	30.99 ± 2.86
	Ti K	44.55 ± 8.61	67.36 ± 10.79
	Ag K	0.49 ± 0.10	1.64 ± 0.29
Ag@TiO_2_ 2000p	O K	55.70 ± 7.64	31.51 ± 2.77
	Ti K	43.61 ± 8.32	66.15 ± 10.53
	Ag K	0.70 ± 0.16	2.34 ± 0.47

**Figure 6:**
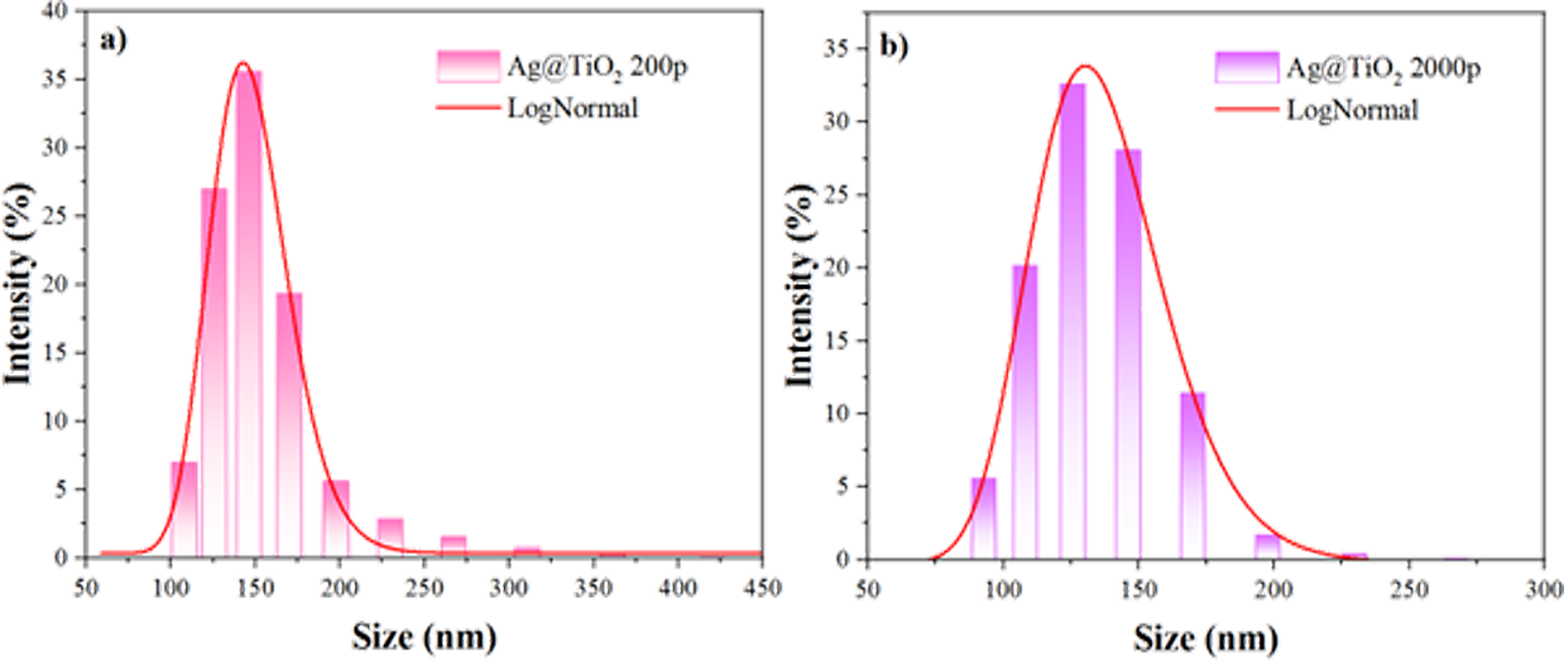
Particle size distribution of Ag@TiO_2_ nanoparticles determined by dynamic light scattering (DLS). (a) Ag@TiO_2_ 200p and (b) Ag@TiO_2_ 2000p NPs.

The nanoparticle sizes and size distributions were measured using a zeta potential analyzer with dynamic light scattering (DLS). Table 2 summarizes the average dimensions of the synthesized nanoparticles. Ag@TiO_2_ 200p and Ag@TiO_2_ 2000p NPs exhibited average hydrodynamic diameters of 145.2 and 134.6 nm, and the size of the nanoparticles averaged in the range of 108–420 and 93–267 nm, respectively (Figure 6). These values are in reasonable agreement with the TEM-derived particle sizes, considering that DLS measures hydrodynamic diameters of particle agglomerates in suspension. The zeta potentials were −13.82 and −14.09 mV, and the electrophoretic mobilities were −1.083 and −1.104 µm·cm·V^−1^·s^−1^, respectively. The zeta potential reveals that the nanoparticles possess a negative surface charge, and the magnitude suggests they are in a state of initial instability.

**Table 2:** Average hydrodynamic diameter, zeta potential, and electrophoretic mobility of Ag@TiO_2_ 200p and Ag@TiO_2_ 2000p NPs at pH 2.4.

Sample	*D* (nm)	Zeta potential (mV)	Electrophoretic mobility (µm·cm·V^−1^·s^−1^)

Ag@TiO_2_ 200 p	145.2	−13.82	−1.083
Ag@TiO_2_ 2000 p	134.6	−14.09	−1.104

### Influence of Ag on the bandgap energy of TiO_2_

**Figure 7:**
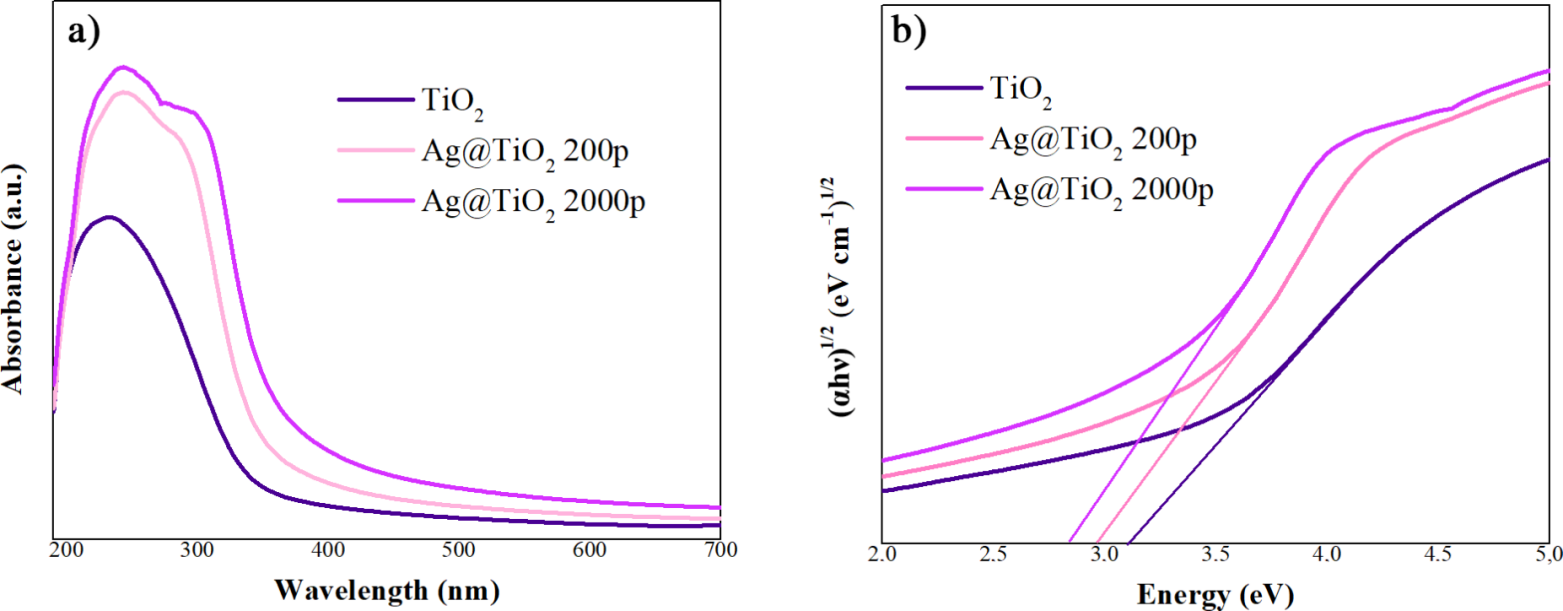
(a) UV–vis absorbance spectra and (b) bandgap energy of TiO_2_ and Ag@TiO_2_ NPs samples.

UV–vis spectroscopy was employed to investigate the effect of Ag doping on TiO_2_ and to determine the bandgaps of both TiO_2_ and Ag@TiO_2_ samples. Figure 7a displays the absorption spectra of TiO_2_ and Ag-modified TiO_2_ samples. These absorption spectra showed that, in contrast to TiO_2_, Ag@TiO_2_ samples showed broadening of the absorption peak due to the addition of Ag. The optical bandgap energy of the prepared samples was calculated with the Tauc model, according to Equation 1:


[1] $$\alpha h\nu =A{\left(h\nu -E_{\text{g}}\right)}^{n},$$

where α is the absorption coefficient, *h*ν is the photon energy, *E*_g_ is the bandgap energy, *A* is a constant, and *n* corresponds to the nature of the transition (i.e., direct or indirect) [23]. A decrease in bandgap energy results in a small redshift in light absorption, as presented in Figure 7b. This resulted in a reduction in the bandgap energy for all of the modified samples, from 3.12 eV for TiO_2_ to 3.08 and 2.97 eV for Ag@TiO_2_ 200p and Ag@TiO_2_ 2000p, respectively. The valence band (VB) and conduction band (CB) edge positions were estimated using the absolute electronegativity method [24–26] combined with the optical bandgap obtained from UV–vis spectroscopy. The CB edge potential, *E*_CB_, was calculated using Equation 2:


[2] $$E_{\text{CB}}=\chi -E_{\text{e}}-0.5E_{\text{g}},$$

where χ is the absolute electronegativity of TiO_2_ (5.81 eV) [25] and *E*_e_ is the energy of free electrons on the hydrogen scale (4.5 eV) [26].

The VB edge position, *E*_VB_, was obtained from Equation 3:


[3] $$E_{\text{VB}}=E_{\text{CB}}+E_{\text{g}}.$$

As shown in Table 3, modification of TiO_2_ with Ag, leads to a gradual narrowing of the bandgap and slight shifts of both CB and VB edge positions. The bandgap decreases from 3.12 eV for bare TiO_2_ to 2.97 eV for Ag@TiO_2_ 2000p, indicating an enhanced visible-light absorption capability. The CB edge shifts slightly toward more positive potential with increasing Ag content, which can be attributed to the interaction between Ag species and the TiO_2_ lattice as well as the formation of localized energy states near the conduction band. Meanwhile, the VB edge also shows a small downward shift. These changes in band structure facilitate improved separation of photogenerated charge carriers and extend light absorption toward the visible region, which together contribute to the enhanced photocatalytic performance of Ag@TiO_2_ compared to bare TiO_2_.

**Table 3:** Estimated values of bandgap and conduction and valence band potentials for TiO_2_, Ag@TiO_2_ 200p, and Ag@TiO_2_ 2000p.

Catalyst	Bandgap	Conduction band	Valence band

TiO_2_	3.12 eV	−0.25 eV	2.87 eV
200 p Ag@TiO_2_	3.08 eV	−0.23 eV	2.85 eV
2000 p Ag@TiO_2_	2.97 eV	−0.18 eV	2.79 eV

Liza et al. [27] showed that TiO_2_ doping with a transition metal such as Ag may result in the creation of new electronic states in the bandgap, demonstrating that the electron is driven at lower photon energy by a defective state of CB. Additionally, with the addition of Ag, an additional component in the broad band could be noticed in Figure 7a, and it could be associated with Ag nanoparticles. Ag nanoparticles can enhance light absorption due to plasmonic effects. Together, these effects contribute to the improved photocatalytic performance observed in this study [28].

### Photodegradation of rhodamine B with Ag@TiO_2_ (200p and 2000p) NPs

To examine the kinetics of continuous photocatalytic removal of RhB dye from aqueous solutions using the Ag@TiO_2_ 200p and Ag@TiO_2_ 2000p samples, pseudo-first order and pseudo-second order kinetic models were utilized (Equation 4 and Equation 5, respectively).


[4] $$-\ln\left(c/c_{0}\right)=k_{1}t,$$

[5] $$1/c=c_{0}+k_{2}t,$$

where *k*_1_ is the first order rate constant, *k*_2_ is the second order rate constant and *c*_0_ and *c* are the RhB concentrations at times *t* = 0 and *t*, respectively.

**Figure 8:**
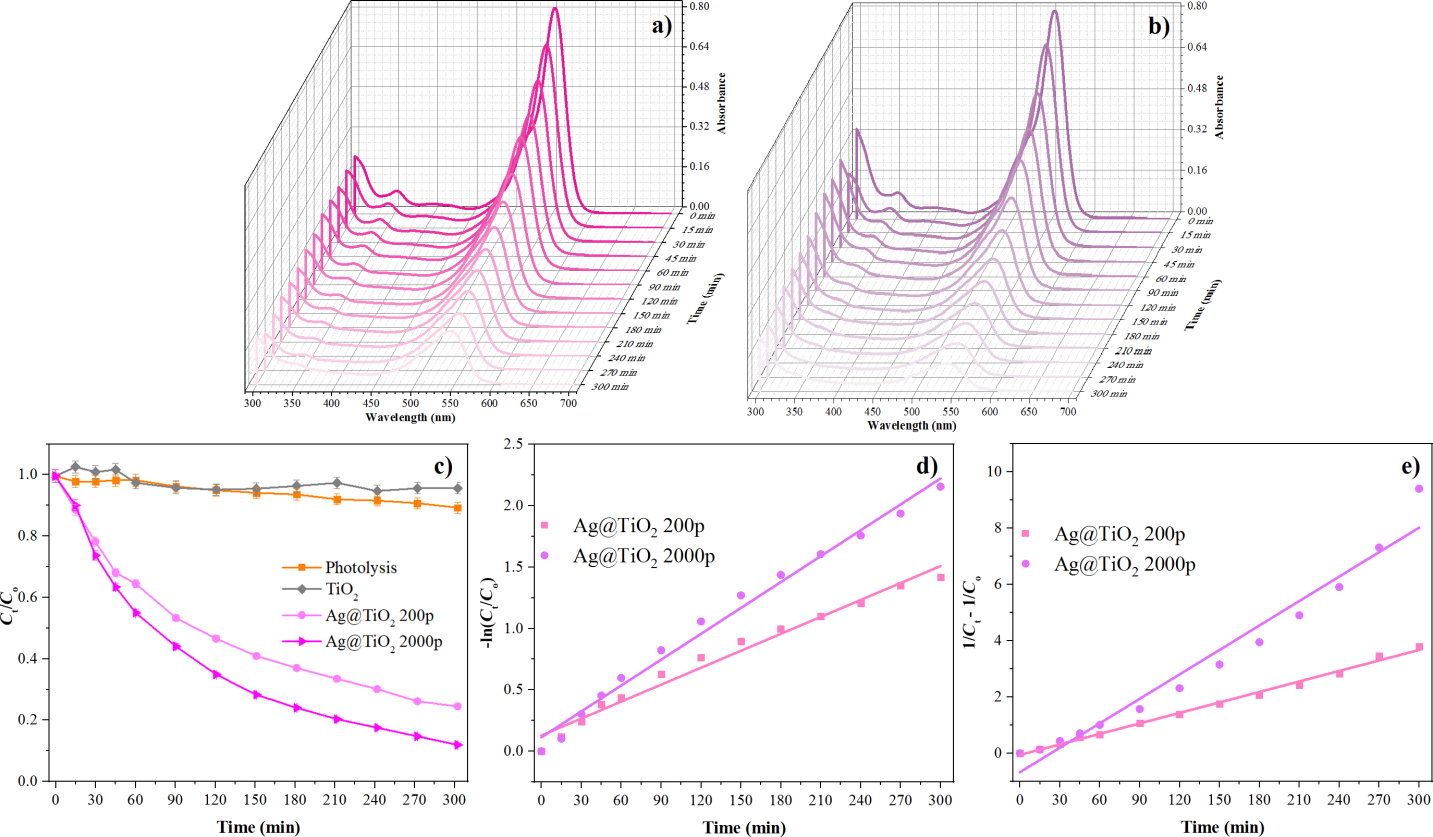
Photocatalytic degradation of RhB with (a) Ag@TiO_2_ 200p NPs and (b) Ag@TiO_2_ 2000p NPs, (c) photocatalytic degradation of RhB, (d) pseudo-first order kinetic model, and (e) pseudo-second order kinetic model for the degradation of RhB.

UV–vis spectroscopy was employed to observe the photocatalytic degradation of RhB, scanned within the range of 300–700 nm. Figure 8a,b presents the UV–visible absorption spectra of RhB in the presence of the synthesized samples as functions of irradiation time with the distinctive absorption peak of RhB at around 553 nm [29]. The photocatalytic capabilities of the synthesized materials were assessed by examining their capacity to degrade a 5 ppm aqueous solution of RhB, utilized as a model organic pollutant, for a time period of 300 min (Figure 8c). The photocatalytic degradation of RhB without a photocatalyst was minimal after 300 min of exposure to UV irradiation using a 300 W Osram Ultra-Vitalux lamp. Similar results were obtained for PLAL TiO_2_ nanoparticles. These results are correlated to the amount of the ablated material in these three samples. As shown in Figure 6a, and based on the Beer–Lambert law, the concentration of TiO_2_ is significantly smaller in samples when pure Ti is ablated. Sample Ag@TiO_2_ 200p NPs exhibited a RhB degradation of 75.8%, while Ag@TiO_2_ 2000p NPs demonstrated a RhB degradation of 88.4%. The experimental results indicate that the correlation coefficient for the pseudo-second order kinetic model exceeds that of the pseudo-first order kinetic model for the Ag@TiO_2_ 200p NPs sample. This suggests that the kinetic behavior of the photocatalytic process in the degradation of RhB can be rather described by the pseudo-second order kinetic model. However, for the Ag@TiO_2_ 2000p NPs sample, the scenario is inverted, leading to the conclusion that the photocatalytic degradation is more accurately represented by the first order kinetic model (Figure 8d,e). The rate constants and their respective correlation values (*R*^2^(1) and *R*^2^(2)) were calculated from the plots and are presented in Table 4.

**Table 4:** Values of the pseudo-first and pseudo-second order rate constants and the corresponding correlation coefficients for the photocatalytic degradation of RhB.

Photocatalyst	*k*_1_ (min^−1^)	*R*^2^(1)	*k*_2_ (L·mg^−1^·min^−1^)	*R*^2^(2)

Ag@TiO_2_ 200p	0.0046	0.978	0.0124	0.995
Ag@TiO_2_ 2000p	0.0070	0.988	0.0289	0.963

Both kinetic models show a correlation over 0.96 for both samples. For Ag@TiO_2_ 200p NPs, the data fit better with the pseudo-second order model, indicating that the degradation rate is strongly influenced by the adsorption capacity of the photocatalyst and the concentration of active site. This is in line with the correlation between particle size and silver content, where smaller particles provide a higher surface-to-volume ratio, making surface effects more dominant. In contrast, Ag@TiO_2_ 2000p NPs followed the pseudo-first-order model, which is more typical when the main process is controlled by light absorption and charge carrier transfer rather than surface adsorption. The smaller particle size and higher silver content in the 2000p sample improved electron–hole separation; hence, the reaction was mainly driven by photocatalytic activity. The improved activity of Ag@TiO_2_ is not mainly due to bandgap reduction. It is more likely related to Ag–TiO_2_ interaction, where Ag reduces electron–hole recombination and enhances light absorption through surface plasmon resonance, leading to increased formation of reactive oxygen species [28]. These combined effects lead to faster degradation of rhodamine B compared to pure TiO_2_. The different kinetic behavior of the samples reflects differences in particle size and Ag surface modification. The kinetic models are used as empirical descriptions of the overall process rather than evidence of distinct mechanisms [30]. Similar trends, where particle size and silver loading influence the kinetic model of the reaction, have also been reported for Ag-TiO_2_ composites in recent studies [16].

### Comparation of current research with literature data

The photocatalytic efficiency of the synthesized Ag@TiO_2_ 200p and Ag@TiO_2_ 2000p nanomaterials was compared with relevant literature data to evaluate the effectiveness of the applied laser synthesis method. In the experiments conducted in this study, at an initial rhodamine B concentration of 5 mg·L^−1^ and UV irradiation power of 300 W for 300 min, a degradation efficiency of 75.8% was achieved for Ag@TiO_2_ 200p NPs and 88.4% for Ag@TiO_2_ 2000p NPs.

Table 5 provides a qualitative comparison of reported photocatalytic efficiencies to contextualize our results, acknowledging that different experimental conditions were used in the literature. Although maximum degradation efficiencies of up to 100% (Table 5) have been reported in the studies by Hussain et al. [31] and Liang et al. [20], these results were typically obtained using longer irradiation times, higher initial RhB concentrations (10 mg·L^−1^), different synthesis methods (e.g., UV-assisted photoreduction, hydrothermal synthesis, and microwave synthesis), more powerful light sources (500 W), and higher dosages of the photocatalyst. It is important to note that the samples in the present study were prepared using a purely physical method, that is, a combination of pulsed laser deposition and ablation without the use of chemical reducing agents or stabilizers, making this approach more environmentally friendly.

**Table 5:** Comparison of the results obtained with literature data.

Catalyst and dosage	Initial RhB concentration (mg·L^−1^)	Synthesis method	Irradiation parameters	Time (min)	Removal efficiency (%)	Reference

Ag@TiO_2_ 200p 0.098 mg·mL^−1^	5	pulsed laser deposition + laser ablation	300 W UV-B (280–315 nm) UV-A (315–400 nm)	300	75.8	this work
Ag@TiO_2_ 2000p 0.1 mg·mL^−1^	5	pulsed laser deposition + laser ablation	300 W UV-B (280–315 nm) UV-A (315–400 nm)	300	88.4	this work
Ag/TiO_2_-I 2 mg·mL^−1^	10	UV-assisted photoreduction (without calcination)	vis, Xe lamp (500 W)	180	100	[20]
Ag/TiO_2_-I 2 mg·mL^−1^	10	UV-assisted photoreduction (without calcination)	UV, high-pressure mercury lamp (500 W, λ = 365 nm)	180	100	[20]
Ag/TiO_2_-II 2 mg·mL^−1^	10	UV-assisted photoreduction (with calcination)	vis, 500 W Xe lamp	180	100	[20]
Ag/TiO_2_-II 2 mg·mL^−1^	10	UV-assisted photoreduction (with calcination)	UV, high-pressure mercury lamp (500 W, λ = 365 nm)	180	100	[20]
Ag–TiO_2_ (1 wt % Ag) 1 mg·mL^−1^	10	formaldehyde-assisted microwave synthesis	UV–vis	60	99	[33]
Ag–TiO_2_ (0.5 wt % Ag) 1 mg·mL^−1^	10	photodeposition synthesis	UV–vis	60	99	[33]
Ag–TiO_2_ (6 wt % Ag) 2 mg·mL^−1^	100	green synthesis	UV	120	90	[35]
Ag–TiO_2_ (6 wt % Ag) 2 mg·mL^−1^	200	green synthesis	UV	120	74	[35]
Ag–TiO_2_ (6 wt % Ag) 2 mg·mL^−1^	300	green synthesis	UV	120	58	[35]
Ag-TiO_2_ 0.5 mg·mL^−1^	4.79	hydrothermal and co-precipitation	UV, Xe lamp (300 W)	14	100	[31]
Ag-1.5/TiO_2_ 1.44 mg·mL^−1^	4.79	γ-irradiation deposition of Ag on TiO_2_	vis, halogen lamp (150 W, λ > 400 nm).	63.8	82.61	[32]
(0.05 M)Ag@TiO_2_ 1 mg·mL^−1^	10	electrospinning + sol-gel infiltration + thermal annealing + photodeposition	vis (360 W)	60	95.5	[34]
Ag/TiO_2_ (1 g·L^−1^) 1 mg·mL^−1^	10	sol–gel + chemical reduction	UV-C (λ = 254 nm).	120	77	[36]
Ag1.5%/TiO_2_ 2 mg·mL^−1^	4.79	γ‑irradiation deposition of Ag on TiO_2_	vis	120	95.59	[37]

Compared to studies that employed lower irradiation intensities (e.g., 160 min by Nhu et al. [32]) and shorter exposure times (e.g., 60 min in the studies by Nyankson et al. [33] and Wang et al. [34]), the efficiency of the Ag@TiO_2_ 2000p NPs sample shows comparable or better degradation performance, despite differences in functionalization approaches and often higher silver content in the literature. Compared to green-synthesized Ag-TiO_2_ samples with a higher silver content (6% Ag) reported by Saeed et al. [35], our Ag@TiO_2_ photocatalysts demonstrate high degradation efficiency (88.4%) despite a significantly lower silver loading. This suggests that the laser-based synthesis provides highly active surface sites, ensuring efficient pollutant removal even with minimal noble metal content.

These findings indicate that the laser-synthesized Ag@TiO_2_ materials, particularly the 2000-pulse sample, exhibit competitive photocatalytic performance compared to more complex or chemically intensive methods reported in the literature. The better efficiency of the 2000p sample can be explained by several factors. The higher amount of silver increases plasmonic effects and helps to separate electrons and holes, reducing recombination. The smaller particle size gives more active surface sites, while the more uniform distribution of Ag in TiO_2_ improves interaction with RhB molecules. Together, these effects allow the 2000p material to use light more effectively and degrade RhB faster than the 200p sample. Furthermore, the difference in efficiency between the 200p and 2000p NPs samples confirms the importance of controlling the number of laser pulses to optimize the structure and activity of Ag@TiO_2_ composites, which is in agreement with previous reports on laser-synthesized Ag-TiO_2_ systems [16].

## Conclusion

This study confirms that dual-laser synthesis (PLD combined with PLAL) effectively produces high-purity Ag@TiO_2_ nanoparticles with a crystalline anatase phase and spherical morphology. The incorporation of Ag leads to a significant reduction in the optical bandgap (down to 2.97 eV), which directly correlates with enhanced photocatalytic performance. The most efficient sample, containing 0.413 wt % of Ag, achieved 88.4% degradation of rhodamine B under UV light. These results highlight the potential of this chemical-free, laser-based approach as a sustainable and versatile platform for fabricating advanced nanomaterials tailored for high-efficiency environmental remediation.

## Experimental

### Synthesis of photocatalysts TiO_2_ and Ag@TiO_2_ (200p and 2000 p)

**Figure 9:**
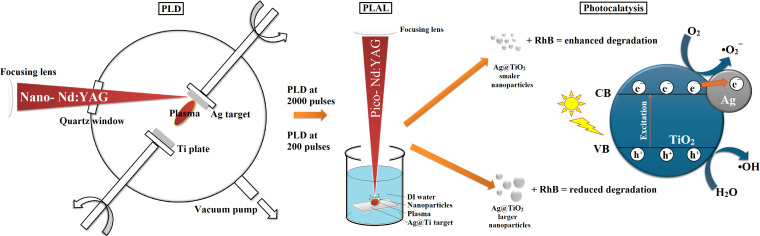
Schematic of the experimental setup.

Thin films of Ag were deposited onto titanium substrates (purity > 99.9%, ThermoScientific) using nanosecond pulsed laser deposition in a vacuum. A 1 mm thick Ag plate (purity > 99.99%, GoodFellow) served as the laser target, with both the Ti substrate and Ag target rotating to ensure uniform film formation. An Nd:YAG laser (1064 nm, 300 mJ, 5 Hz, 5 ns) was focused onto the Ag target with a fluence of 12 J·cm^−2^. Different silver film thicknesses were obtained by varying the number of laser pulses (200 and 2000). Subsequently, the Ag-coated Ti plates and a pure Ti substrate underwent picosecond pulsed laser ablation in Milli-Q water using a Nd:YAG laser (1064 nm, 20 mJ, 10 Hz, 150 ps) to generate Ag-modified TiO_2_ colloidal nanoparticles. The ablation was performed at a laser fluence of approximately 2 J·cm^−2^ for both samples, Ag@TiO_2_ 200p and Ag@TiO_2_ 2000p [21–22]. The experimental setup is shown in Figure 9. Obtained suspensions were murky white with a hint of blue.

### Photolysis of rhodamine B

An 18 mL solution of rhodamine B (5 ppm) was stirred in the dark for 30 min, after which the sample was irradiated with a 300 W Osram Ultra-Vitalux lamp (Osram, Munich, Germany, UV-B (280–315 nm) UV-A (315–400 nm)) for 5 h. The light intensity at the sample surface was measured to be 700 W·m^−2^. Aliquots were taken every 15 min and analyzed using a UV–vis spectrophotometer (LLG Labware, Detroit, MI, USA) over the wavelength range of 300–700 nm, with absorbance monitored at 550 nm.

### Photodegradation of rhodamine B with TiO_2_ and Ag@TiO_2_ (200p and 2000p)

A volume of 2 mL of suspended nanoparticles (TiO_2_, Ag@TiO_2_ 200p, or Ag@TiO_2_ 2000p) catalyst was added to 18 mL of a rhodamine B solution (5 ppm). The mixture was stirred (300 rpm) in the dark for 30 min and then irradiated using a 300 W Osram Ultra-Vitalux lamp (Osram, Munich, Germany) for 5 h. The light intensity at the surface of the reaction mixture was measured at 700 W·m^−2^. Aliquots were taken at 0, 15, 30, 45, 60, 90, 120, 150, 180, 210, and 240 min, centrifuged (2 min, 5000 rpm), and the supernatant was analyzed using a UV–vis spectrophotometer (LLG Labware, Detroit, MI, USA) over the wavelength range of 300–700 nm, with absorbance monitored at 550 nm.

### SEM analysis

A field-emission scanning electron microscope (FESEM), Scios 2 DualBeam (Thermo Fisher Scientific, Waltham, MA, USA), was used to determine the morphology and chemical composition, including surface chemical mapping of samples. The operating voltage was 30 kV. Samples were analyzed without any additional preparation.

### TEM analysis

A FEI Talos F200X microscope (Thermo Fisher Scientific, Waltham, MA, USA) equipped with an X-FEG source and a maximum accelerating voltage of 200 kV was used for microstructural examination utilizing transmission electron microscopy. Investigations were conducted using scanning transmission electron microscopy, high-angle annular dark-field with energy-dispersive spectroscopy, and conventional and high-resolution transmission electron microscopy. A drop of the suspended photocatalyst (Ag@TiO_2_ 200p or Ag@TiO_2_ 2000p) was deposited onto a carbon-coated copper grid and left to dry under ambient conditions. ImageJ software, version 1.54 g, was used for image analysis and interplanar distance computations.

### Measurements of particle size distribution, zeta potential, and electrophoretic mobility

Hydrodynamic size distribution and zeta potential of the nanoparticles were measured using a Zetasizer Ultra (Malvern Panalytical, Malvern, Worcestershire, UK). Disposable plastic cuvettes were employed for size distribution analysis, while capillary cells were used for zeta potential and electrophoretic mobility measurements. Before sample analysis, the nanoparticle colloid solutions were ultrasonicated. This way, all possible agglomerates were broken down into smaller and more uniform particles. Samples were analyzed at the native pH value.

## Data Availability

Data generated and analyzed during this study is available from the corresponding author upon reasonable request.
